# Development and Validation of an ELISA to Detect Antibody to *Onchocerca volvulus* Infection Using Mammalian Expressed Recombinant Ov16

**DOI:** 10.4269/ajtmh.25-0725

**Published:** 2026-04-07

**Authors:** Sylvia Ossai, Yong Wang, Eric S. Elder, Karana Wickens, Kimberly Y. Won, W. Evan Secor, Sukwan Handali

**Affiliations:** ^1^ CDC Foundation, Atlanta, Georgia;; ^2^ Synergy America, Inc., Duluth, Georgia, USA;; ^3^ Laboratory Science and Diagnostics Branch, Division of Parasitic Diseases and Malaria, Centers for Disease Control and Prevention, Atlanta, Georgia, USA

## Abstract

Programs designed to eliminate *Onchocerca volvulus* transmission rely on measurement of Ov16-specific antibodies in children <10 years old and molecular detection of parasites in blackfly vectors. Antibody testing can be conducted using rapid diagnostic tests or an ELISA, depending on location of testing and sample throughput needs. In previous efforts to establish anti-Ov16 testing in endemic country laboratories using existing tests, we encountered difficulties with assay reproducibility, reagent availability and shipping, and user friendliness. To address these challenges, we developed and validated an ELISA using Ov16 recombinant antigen expressed in a mammalian protein expression system that can be completed within 2 hours and does not require refrigeration during shipment. The assay was developed and validated using two distinct sets of dried blood spot samples that included defined positive, negative, and samples from infections with potential cross-reactivity. During development, the ELISA had 92.98% (95% CI = 88.28–97.68%) sensitivity and a specificity of 98.50% (95% CI = 93.80–100%). The cutoff point established during development was then applied to the validation set of specimens, which demonstrated a sensitivity of 89.47% (95% CI = 84.77–94.17%) and 98.32% (95% CI = 93.62–100%) specificity. When repeated over 4 days by 2 different operators, all replicates yielded reproducible results within a coefficient of variance of 20%. In addition, we performed stability testing on assay reagents to evaluate their suitability for shipping at ambient temperatures. The resulting assay will be useful for monitoring anti-Ov16 prevalence in endemic country laboratories.

## INTRODUCTION

Onchocerciasis, also known as river blindness, is a neglected tropical disease (NTD) caused by infection with the filarial nematode *Onchocerca volvulus*. It affects ∼21 million people, with 99% of cases reported in 31 sub-Saharan countries.^1^ It is a high-burden disease in humans that poses a major public health concern and is targeted for elimination.^2^ In addition to blindness, morbidity caused by onchocerciasis can include severe itching, dermatitis,^3^ neurological disorders resembling epilepsy,^4^ nodding syndrome, and stunted growth.^5^ Because of the public health burden caused by onchocerciasis, annual or semiannual mass drug administration (MDA) of the antihelminthic drug ivermectin has been used to control and eventually eliminate *O. volvulus* transmission.^6^ Although skin snips are useful for detecting active human infection, they become less sensitive in areas approaching elimination. As a result, guidelines for monitoring and evaluation of MDA programs recommend measuring prevalence of IgG4 antibodies to *O. volvulus* in children under 10 years old to assess transmission.^6^ The Ov16 antigen, which is present in all stages of the parasite’s life cycle,^7^ is a highly sensitive and specific marker for detecting IgG4 antibodies in individuals previously exposed to onchocerciasis.^8^^,^^9^ Currently, in program settings, anti-Ov16 antibodies are detected by rapid diagnostic tests or ELISA; the choice of test format depends on the context in which testing is needed.^10^^–^^12^

Various laboratory-based Ov16 ELISA formats have been developed for programmatic use in onchocerciasis endemic countries, including the version implemented by the Onchocerciasis Elimination Program for the Americas (OEPA). However, the OEPA ELISA has several limitations, such as low sensitivity,^13^ the absence of standardized assay controls, and cross-reactivity with other helminths such as *Wuchereria bancrofti* and *Brugia malayi*, which can potentially lead to false-positive results.^14^ These factors have led to inconsistent assay results between laboratories, resulting in greater variability both within and across testing environments. The OEPA ELISA incorporates some reagents produced in a university laboratory, making it difficult to produce in large quantities and deploy in remote, resource-limited settings.^6^ The reagents used for the ELISA often need to be stored and transported under specific cold temperature conditions, which can often result in importation issues and variability, specifically in tropical or rural areas.^6^^,^^15^ In addition, the assay is time-consuming with low throughput, because it typically requires several steps and takes approximately 7 hours to process, slowing down large-scale screening efforts.^13^ A commercially produced Ov16 ELISA is available (https://www.globalpointofcare.abbott/ww/en/product-details/bioline-onchocerciasis-igg4-elisa.html)^12^; however, it likewise requires a cold chain during shipment and is only available for purchase in quantities that often exceed a country’s needs. Neither of these tests meets the target product profiles (TPPs) for antibody detection developed by the onchocerciasis diagnostic technical advisory subgroup.^6^^,^^16^^,^^17^ Further, when we attempted to assist endemic countries to establish these tests in their laboratories, we observed problems with assay precision and reproducibility, rendering the results needed for ongoing MDA decisions unreliable.

To address these challenges, we developed an Ov16 ELISA with the aim of meeting parameters outlined in the TPPs.^16^^,^^17^ Major changes to the ELISA included 1) using a mammalian-expressed recombinant antigen to avoid potential cross-reactivity to any contaminating bacterial proteins; 2) optimizing the conditions for coating, blocking, and drying plates, to enable large batch production and ensure plate consistency; 3) replacing the OEPA ELISA’s two-step avidin-biotin alkaline phosphatase development system with a single step using horseradish peroxidase (HRP) conjugated secondary antibody; 4) implementation of standardized controls; and 5) development and evaluation of assay components that can be shipped at ambient temperature, eliminating the need for shipment under cold conditions.

## MATERIALS AND METHODS

### Human subjects.

Residual onchocerciasis serum specimens were de-identified and used under Protocol No. 6756, approved by the CDC Institutional Review Board. This activity was reviewed by the CDC; because researchers evaluating specimens had no access to personal identifiers, they were considered not engaged in human subjects research. The study was conducted consistent with applicable federal law and CDC policy (45 C.F.R. part 46.102(l)(2), 21 C.F.R. part 56; 42 U.S.C. Sect. 241(d); 5 U.S.C. Sect. 552a; 44 U.S.C. Sect. 3501 et seq.).

### Serum sample sets.

Two distinct sets of samples were used to qualify and validate the assay (Table 1). For assay development and optimization, including determination of the assay cutoff, the sensitivity panel consisted of contrived dried blood spots (DBS) using sera or plasma obtained from individuals with parasitology-confirmed onchocerciasis living in sub-Saharan Africa (*n* = 80)^18^ and Guatemala (*n* = 34).^19^^,^^20^ Contrived DBS were made by first removing the serum from whole blood obtained from uninfected donors and then washing the red blood cells three times with 1 × phosphate buffered saline (PBS) by centrifugation. The washed, serum-free red blood cells were then mixed with an equal volume of test sera, and 10 *µ*L of this mixture was spotted onto TropBio Filter Paper disks (Cellabs, Sydney, Australia, Cat. no. 05-002-12), the same filter paper used for field-collected DBS. The specificity panel included *O. volvulus* negative DBS samples (*n* = 80) from individuals living in Haiti and the Philippines where onchocerciasis is not endemic.^6^ The specificity panel was augmented by contrived DBS made using 31 sera from Haitian individuals with *W. bancrofti* infections and 12 sera from Cameroonians with *Loa loa* infection.^21^ Eighty-nine sera from persons with other parasitic infections such as schistosomes (*Schistosoma mansoni*, *S. haematobium*, and *S. japonicum*), *Clonorchis sinensis*, intestinal protozoa, or soil-transmitted helminths from nonendemic regions were also used to make contrived DBS for specificity testing.

**Table 1:** Independent sample sets used for assay development and validation

Sample	DBS Type	Development (*n/N*)	Validation (*n/N*)
*O. volvulus* confirmed	Contrived	106/114	102/114
*O. volvulus* negative	Field collected	0/80	0/38
*Wuchereria bancrofti*	Contrived	0/31	0/99
*Loa loa*	Contrived	0/12	0/12
*Entamoeba histolytica*	Contrived	0/1	0/3
*Hymenolepis nana*	Contrived	0/1	0/2
*Trichinella spiralis*	Contrived	0/2	0/2
*Clonorchis sinensis*	Contrived	0/6	0/6
*Schistosoma mansoni*	Contrived	0/13	2/10
*Schistosoma haematobium*	Contrived	0/10	0/10
*Schistosoma japonicum*	Contrived	3/10	1/10
*Trichuris trichiura*	Contrived	0/7	0/13
*Necator americanus*	Contrived	0/7	0/9
*Ascaris lumbricoides*	Contrived	0/10	1/14
Type 1 Diabetes	Contrived	0/5	0/5
Rheumatoid Factor	Contrived	0/5	0/5

*n* = number positive; *N* = number tested.

The assay was validated using panels of DBS samples from the same geographic regions as the ones used for development but were from different individuals. The validation sensitivity panel consisted of 114 contrived DBS samples from individuals with onchocerciasis, and the specificity panel included 250 contrived DBS samples from individuals from areas nonendemic for onchocerciasis who had other helminth parasite infections.

### Recombinant protein synthesis, expression, and purification.

The encoded gene of the OVOC12871OV-16 protein was expressed as a fusion protein with *Schistosoma japonicum* glutathione-S-transferase (GST) in both bacterial and mammalian expression systems. For recombinant bacterial expression (rOv16b), the gene was inserted into the pGS21a expression vector (GenScript, Piscataway, NJ), which was then transformed into One Shot BL21 (DE3) chemically competent *E. coli* (Invitrogen, Carlsbad, CA, Cat. no. C601003). Proteins were expressed in 1L cultures induced with isopropyl β-d1-thiogalactopyranoside (IPTG) at 37°C for 3 hours, then harvested by centrifugation and suspended in lysis buffer with protease inhibitor cocktail (ThermoFisher Scientific, Waltham, MA, Cat. no. PI78438). The pellet was stored at −80°C, thawed and treated with PBS/Triton-X 100, and lysozyme followed by sonication. The mixture was centrifuged, and the supernatant was filtered. The recombinant GST fusion protein was purified using glutathione Sepharose 4B affinity chromatography column (GE Healthcare, Chicago, IL). For recombinant mammalian Ov16 (rOv16m), we contracted GenScript to subclone the gene into a Chinese Hamster Ovary (CHO) expression vector, produce and purify the antigen, and provide a certificate of analysis (CoA) for each lot. Protein concentrations were measured using the Bradford assay employing a protein standard (Millipore Sigma, Burlington, MA, Cat. no. 11-100-5057) and a protein assay dye reagent concentrate (Bio-Rad Laboratories, Hercules, CA, Cat. no. 5000006).

### SDS-PAGE separation of the recombinant protein.

Immunoblots were used to identify and characterize the purified recombinant proteins based on size and antibody reactivity.^22^ In brief, 0.05 mg of the recombinant protein were treated with 10% sodium dodecyl sulfate (SDS) (ThermoFisher Scientific, Cat. no. PI28312) and heated in a water bath at 65°C for 15 minutes, then mixed with 6% glycerol (ThermoFisher Scientific, Cat. no. BP229-4). The protein mixture underwent 1-D SDS-PAGE on Mini-PROTEAN TGX Precast Gels 4–20% (Bio-Rad Laboratories, Cat. no. 456-1096) for 30 minutes followed by transblotting onto a nitrocellulose membrane (Bio-Rad Laboratories, Cat. no. 1704159) for 7 minutes. The membranes were washed with PBS containing 0.3% Tween-20 and incubated overnight at 4°C with a 1:100 dilution of pooled test sera (negative control, *O. volvulus*–positive, or *W. bancrofti–*pooled positive) in PBS with 0.3% Tween-20 and 5% nonfat dry skim milk. Bound antibodies were detected using a 1:1,000 dilution of mouse antihuman IgG4 Fc (clone HP6025) conjugated to horseradish peroxidase (Southern Biotech, Birmingham, AL, Cat. no. 9200-05). Likewise, mouse monoclonal anti-GST (GenScript, Cat. no. A00866) was used to confirm that the GST-tagged fusion proteins had successfully transferred to the membrane. After a 1-hour incubation at room temperature, the blots were developed using 3, 3′-diaminobenzidine (DAB) chromogenic substrate for peroxidase (ThermoFisher Scientific, Cat. no. 34002) to visualize the protein profile.

### ELISA development.

The ELISA using rOv16m was optimized using 96-well Immulon 2HB plates (Thermo Scientific, Cat. no. 3455) with *O. volvulus*–positive and *O. volvulus*–negative controls. A checkerboard titration was performed with 2-fold serial dilutions of the antigen, serum, and secondary antibody. Antigen concentrations ranged from 10 *µ*g/mL to 0.01 *µ*g/mL, serum dilutions from 1:50 to 1:400 and HRP-conjugated HP6025 dilutions from 1:500 to 1:8,000. The assay’s development time was evaluated using a kinetic curve, with absorbance readings at 650 nm collected every 30 seconds for 15 minutes without stopping the reaction using a VersaMax Kinetic Microplate Reader (Molecular Devices, San Jose, CA) after the addition of SureBlue 3,3′,5,5′-Tetramethylbenzidine(TMB) substrate (KPL, Gaithersburg, MD, Cat. no. 520000). Optimal conditions were determined based on the signal-to-noise ratio between controls.

### Coating and drying antigen on the plate.

To address a common cause of ELISA variability, we developed a standardized protocol for coating, blocking, and drying large batches of plates. Wells were sensitized with 100 *µ*L/well of 2.5 *µ*g/mL antigen in coating buffer (50 mM Tris-HCl, pH 8, 2 mM EDTA, 0.3 M KCl) and incubated overnight at 4°C. The plates were washed three times with wash buffer (PBS containing 0.3% Tween-20). Then, 100 *µ*L/well of StabilCoat (Surmodics, Eden Prairie, MN, Cat. no. SC01) was added for blocking, and the plates were incubated for 30 minutes with shaking at room temperature (15–25°C). After discarding the blocking buffer, the plates were vacuum-dried at 30°C for 4 hours in a vacuum oven (Cascade TEK, Plano, TX). Each plate was then sealed in an aluminum foil pouch with desiccant. We evaluated the stability of the precoated, dried plates by exposing them to various temperatures prior to evaluation of their performance: refrigeration temperature (2–8°C), ambient temperature (20–25°C) and incubation temperature (30–45°C) for different lengths of time to simulate different storage and shipping conditions.

### Standard curve.

To better correct for variability between assay runs, a standard curve using a humanized monoclonal IgG4 antibody against Ov16 (AbD19432ie_hIgG4; BioRad AbD Serotec, Puchheim, Germany) was included on each plate. The curve consisted of seven concentrations, ranging from 0.9 ng/mL to 60 ng/mL of the monoclonal antibody, diluted in PBS containing 0.3% Tween-20 and 5% nonfat dry milk solution. This standard curve was included in duplicate on each plate. Assay precision was evaluated by determining the coefficient of variation (CV) of the selected standard curve concentrations across multiple assay plates and users over several days.

### Limit of detection (LOD).

We conducted a range-finding experiment to establish the lower LOD by testing 10-fold serial dilutions of AbD19432ie_hIgG4 at concentrations ranging from 0.01 ng/mL to 10 ng/mL in quadruplicate. Probit analysis of the fraction of positive results at each concentration were used to estimate the assay’s initial LOD. This value was then validated by assaying the initial LOD concentration ≥20 times to confirm at least 95% of the tests yield a positive result.

### Stability of monoclonal antibody controls.

We conducted stability testing on the monoclonal antibody used as the assay standard curve and controls. AbD19432ie_hIgG4 was diluted in defibrinated human plasma basematrix (SeraCare, Milford, MA, Cat. no. 1805-0074), spotted onto TropBio Filter Paper disks, and dried overnight at room temperature (15–25°C). The stability of the spotted filter papers was evaluated under different temperature conditions (−10 to −30°C, 2–8°C, 20–25°C, and 30–45°C) over a 6-month period to simulate different storage and shipping conditions. Baseline values were established using five replicates at each concentration of the monoclonal antibody. These were used to define the predetermined upper and lower limits of the acceptable range, with values exceeding three standard deviations from the baseline considered indicative of instability.

### Optimized ELISA parameters.

Plates and reagents were equilibrated to room temperature (15–25°C). Dried standards and DBS samples were eluted overnight at 2–8°C in sample buffer (PBS with 0.3% Tween-20 and 5% skim dry milk) to achieve a 1:100 final dilution of sera. After incubation and eluted sample equilibration to room temperature, 100 *µ*L of eluted samples were added in duplicate to the plates and incubated for 30 minutes on a plate shaker at room temperature. The plates were washed three times with wash buffer. HRP-conjugated HP6025 was diluted 1:1,000 in sample buffer and 100 *µ*L was added to each well, followed by incubation and washing as described previously. Then, 100 *µ*L of SureBlue TMB Peroxidase Substrate was added and incubated on a plate shaker for 10 minutes at room temperature (15–25°C). The reaction was stopped by adding 100 *µ*L of 1 N H_2_SO_4_ (Fisher Scientific, Fair Lawn, NJ, Cat. no. SA212), and absorbance was measured at 450 nm using the VersaMax Kinetic ELISA Microplate Reader. Softmax Pro 7 (Molecular Devices) was used to create a standard curve and estimate the concentration of each well.

## STATISTICAL ANALYSES

Data were tabulated, and interplate CV and LOD were calculated using Microsoft Excel^®^. Determination of the cutoff value and assay performance metrics including sensitivity and specificity were obtained by using R statistical software (v. 3.5.3; R Foundation for Statistical Computing, Vienna, Austria) along with the pROC package.^23^ In brief, sensitivity was determined as the proportion of samples from persons with onchocerciasis who tested positive in the rOv16m ELISA, and specificity was calculated as the proportion of samples from individuals who did not have *O. volvulus* infection who tested negative. Test accuracy was calculated as the number of specimens correctly identified as positive or negative as a proportion of the total number of samples tested.

## RESULTS

### Recombinant protein expression and purification.

**Figure 1.**
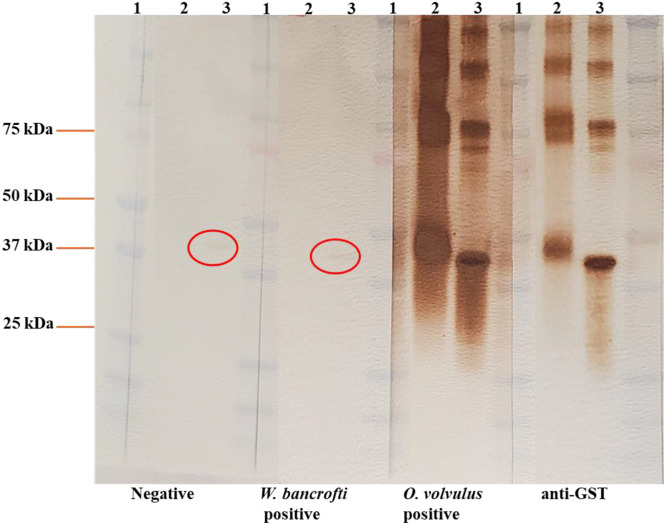
Comparison of rOv16m (mammalian-expressed) and rOv16b (bacterial-expressed) antigens. Proteins were separated using SDS-PAGE and subsequently transferred to a nitrocellulose membrane. The membranes were incubated with sera from pooled negative controls, pooled *O. volvulus*-positive samples, and pooled *Wuchereria bancrofti*–positive samples. Mouse anti-GST antibodies served as a positive control, as rOv16m and rOv16b were expressed as GST fusion proteins. Antigen–antibody complexes were visualized using diaminobenzidine (DAB) as a peroxidase substrate. Lane 1: molecular weight marker; Lane 2: rGST-Ov16m; Lane 3: rGST-Ov16b. Faint cross-reactivities with rGST-Ov16b are circled in red.

The expressed and purified rOv16m (mammalian-expressed) and rOv16b (bacterial-expressed) proteins were tested for reactivity with specific IgG4 using immunoblotting (Figure 1). Pooled sera from individuals with *W. bancrofti* infections or no known helminth infections recognized some bands in the rOv16b preparation but no cross-reactivity with the rOv16m preparation expressed in mammalian cells (lane 2).

### Assay development and cutoff determination.

**Figure 2.**
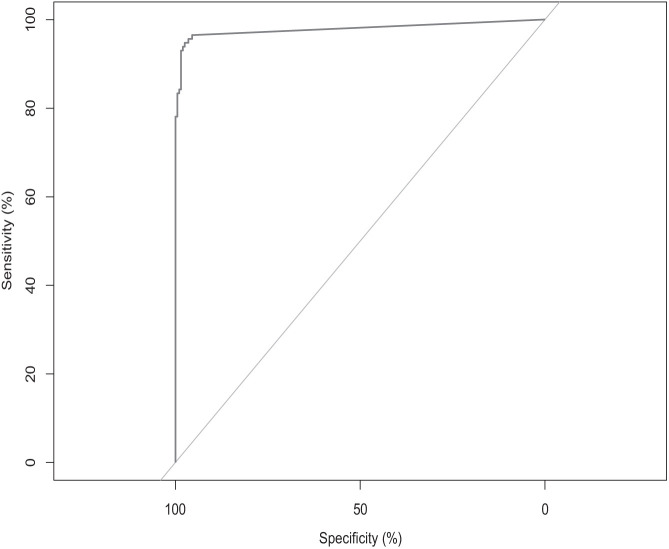
rOv16m ELISA receiver operating characteristic (ROC) curve to establish the assay cutoff. A total of 114 samples from individuals confirmed positive for *O. volvulus* microfilariae by skin snip were included in the sensitivity sample set, and 200 samples from controls or individuals with other helminth infections such as *Wuchereria bancrofti*, *Schistosoma* spp., *Loa loa*, etc., were included in the specificity sample set. ROC analysis indicated the optimal cutoff was 4.7 ng/mL. At this cutoff value, the assay demonstrated a sensitivity of 92.9% (95% CI = 88.2–97.6%) and a specificity of 98.5% (95% CI = 96.8–100%).

The development sensitivity panel included 114 contrived DBS, using samples collected from individuals with parasitology-confirmed *O. volvulus* infections. The specificity panel consisted of 200 DBS, which included 80 natural DBS and 120 contrived DBS, obtained from individuals infected with other helminth parasites or with no known parasitic infections (Table 1). These panels were used to create a receiver operating characteristic (ROC) curve and determine the optimal cutoff threshold for the ELISA (Figure 2). Sample optical densities (ODs) were compared with ODs from the standard curve of the humanized monoclonal antibody. ROC analysis indicated the optimal cutoff was 4.7 ng/mL. At this cutoff value, the assay demonstrated a sensitivity of 92.98% (95% CI = 88.28–97.68%) and a specificity of 98.50% (95% CI = 93.80–100%). There was no reactivity of sera from individuals with *W. bancrofti* or *L. loa*, to rOv16m. However, 3 out of 10 (30%) individuals with *S. japonicum* infections showed reactivity.

### Assay validation.

After the determination of the assay cutoff threshold, internal assay validation using different sets of specimens from individuals who were either positive or negative for *O. volvulus* as described in the section above was conducted to determine assay sensitivity, specificity, accuracy, and precision. The sensitivity of the assay using the validation panel of contrived DBS samples was 89.47% (95% CI = 84.77–94.17%), and the specificity was 98.32% (95% CI = 93.62–100%). Twenty percent (2/10) and 10% (1/10) cross-reactivity were seen with *S. mansoni–* and *S. japonicum*–positive samples, respectively. One out of 14 (7.14%) *A. lumbricoides*–positive samples also showed a positive response. ELISAs performed with rOv16m-GST fusion protein, cleaved and purified rOv16m, and GST suggested that the false-positive sera from persons with schistosome infection were reacting with GST and that the sample from the individual with *Ascaris* infection reacted with rOv16m (Table 2). Immunoblots performed with these sera showed no reactivity of the schistosome samples and a faint but visible reaction with rOv16m for the sample from the person with ascaris infection (data not shown).

**Table 2:** Optical density of false-positive samples with fusion protein, rOv16m alone, and GST alone

Sample	rOv16m with GST	rOv16m No GST	GST Only
*Schistosoma mansoni* 1350	**2.339**	0.083	**0.787**
*S. mansoni* 1352	**2.652**	0.108	**0.778**
*Schistosoma japonicum* 207	**3.318**	0.190	**2.008**
*Ascaris* 20A	**2.971**	**2.960**	0.109
Negative control pool	0.093	0.185	0.161
Ov-positive pool	**4.000**	**3.382**	0.414

Positive results are shown in bold text.

The overall accuracy of the assay was 95.45% (95% CI = 90.75–100%), evaluated using 114 contrived DBS samples spiked with *O. volvulus* and 238 (200 contrived DBS and 38 natural DBS) samples spiked with various other helminth infections, including lymphatic filariasis, schistosomiasis, loiasis, and soil-transmitted helminths. The precision of the assay was assessed by testing 10 positive and 10 negative contrived DBS samples over a 4-day period by two operators. Positive samples were selected to include low to medium positivity. This evaluation demonstrated that all results for a given sample fell within a coefficient of variation (CV) ≤20% among the positive samples (Table 3).

**Table 3:** Average optical density at 450 nm of samples used for precision testing

		P1	P2	P3	P4	P5	P6	P7	P8	P9	P10
Op. 1	Day 1	1.26	1.29	1.48	0.74	1.25	1.83	1.23	2.15	0.92	1.33
	Day 2	1.26	1.22	1.36	0.67	1.13	1.60	1.30	2.06	0.79	1.31
	Day 3	1.18	1.21	1.39	0.70	1.08	1.70	1.16	2.17	0.70	1.08
	Day 4	0.91	1.15	1.50	0.72	1.27	1.70	1.18	2.21	0.84	1.40
Op. 2	Day 1	1.17	1.29	1.66	0.75	1.14	1.53	1.15	2.20	0.81	1.41
	Day 2	0.81	1.12	1.68	0.70	1.28	1.84	1.32	2.13	0.92	1.38
	Day 3	0.82	1.20	1.60	0.78	1.21	1.69	1.24	2.08	0.79	1.14
	Day 4	0.84	1.84	1.54	0.80	1.08	1.64	1.59	2.28	0.85	1.48
	Mean	1.0	1.3	1.5	0.7	1.2	1.7	1.3	2.2	0.8	1.3
	SD	0.2	0.2	0.1	0.0	0.1	0.1	0.1	0.1	0.1	0.1
	% CV	19.88	17.95	7.78	6.03	7.22	6.17	11.26	3.35	8.64	10.52

		N1	N2	N3	N4	N5	N6	N7	N8	N9	N10
Op. 1	Day 1	0.06	0.01	0.09	0.12	0.02	0.11	0.01	0.02	0.03	0.01
	Day 2	0.05	0.00	0.07	0.10	0.02	0.10	0.01	0.02	0.03	0.00
	Day 3	0.05	0.01	0.09	0.13	0.02	0.09	0.01	0.02	0.03	0.01
	Day 4	0.05	0.01	0.09	0.13	0.02	0.10	0.02	0.03	0.03	0.01
Op. 2	Day 1	0.04	0.02	0.07	0.13	0.03	0.09	0.01	0.03	0.03	0.01
	Day 2	0.10	0.03	0.10	0.15	0.03	0.13	0.02	0.03	0.04	0.03
	Day 3	0.06	0.01	0.09	0.13	0.02	0.12	0.01	0.03	0.03	0.02
	Day 4	0.06	0.01	0.09	0.16	0.02	0.12	0.01	0.03	0.04	0.01
	Mean	0.1	0.0	0.1	0.1	0.0	0.1	0.0	0.0	0.0	0.0
	SD	0.0	0.0	0.0	0.0	0.0	0.0	0.0	0.0	0.0	0.0
	% CV	27.29	55.59	12.42	14.78	25.8	13.47	30.35	17.07	14.2	68.87

CV = coefficient of variation; N1 = negative sample 1; N2 = negative sample 2, etc.; Op. 1 = operator 1; Op. 2 = operator 2; P1 = positive sample 1; P2 = positive sample 2, etc.; SD = standard deviation.

### LOD.

Probit analysis of the data from the range-finding experiment estimated the initial LOD as 3.4 ng/mL of the Ov16-specific humanized monoclonal IgG4 antibody. These experiments were conducted on different days by two operators using separate precoated dried plates. To confirm the initial LOD estimate, 24 replicates were tested at this concentration, and all 24 were positive.

### Dried Ov16m plate stability.

**Figure 3.**
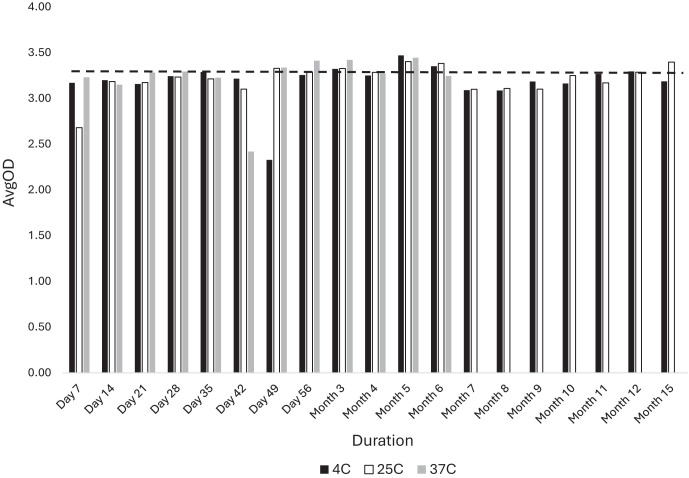
Stability profile of rOv16m coated plates at different temperatures (4°C, 25°C, and 37°C) over a 15-month period. Data points represent the mean OD at 450 nm of 62.5 ng/mL of the humanized anti-Ov16 monoclonal antibody (mAb) plotted over time. The dashed line represents the baseline value on day 0 (3.19).

The coated and dried rOv16m plates were incubated at different temperatures for different lengths of time to evaluate their stability (Figure 3). The freshly precoated, dried plate was evaluated and established as a reference point (day 0) prior to exposure to various temperatures. The plates exhibited stability for up to 15 months at both 4°C and 25°C. In addition, the plates maintained stability throughout a 6-month study period at 37°C and demonstrated integrity for 7 days at 45°C (data not shown). These were the maximum times tested at these higher temperatures because of the low likelihood that plates would be exposed to these higher temperatures for longer periods of time.

### Monoclonal antibody standards stability.

**Figure 4.**
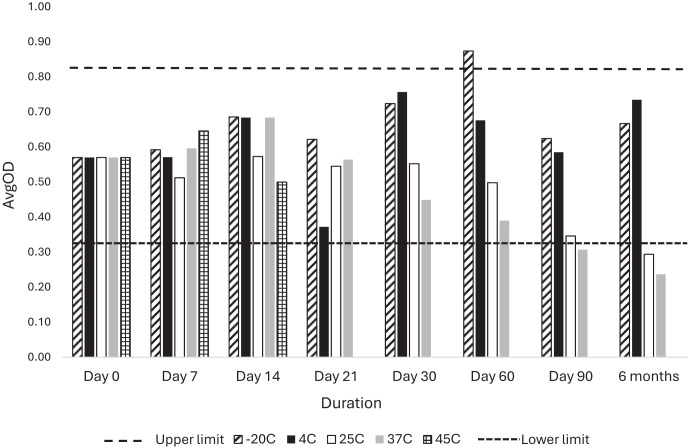
Stability profile of the dried humanized anti-Ov16 mAb stored at different temperatures over a 6-month period. Data points represent the average optical density at 450 nm of one point of the standard curve (7.5 ng/mL) at specified time points in comparison with the predefined upper and lower specification limits.

The monoclonal antibody standards exhibited stability within three standard deviations of the baseline (day 0) when stored at −20°C or 4°C throughout the 6-month study period, whereas a gradual decline was noted for standards stored at 25°C and 37°C, with values becoming unacceptable by 3 months (Figure 4). Standards stored at 45°C were only tested for up to 14 days but retained acceptable activity through this time.

## DISCUSSION

To improve the reliability and usability of serological testing for antibodies to Ov16 for onchocerciasis control programs, we have developed and validated an updated Ov16 ELISA. One of the major changes was to express the antigen in a mammalian cell line instead of using bacterial expression systems. Key differences between bacterial and mammalian cell expression systems affect the folding, post-translational modifications, and functionality of the recombinant proteins. In addition, even after purification of rOv16b, some bacterial antigens may persist, resulting in nonspecific binding (Figure 1), thus potentially decreasing assay specificity. This reactivity is not seen in the lanes with rOv16m. Other changes included producing batches of dried, coated plates that had performance confirmed prior to distribution, thus reducing potential errors that can occur during antigen coating in individual laboratories and promoting greater consistency across laboratories. In the authors’ experiences, the most frequent errors resulting in poor ELISA performance occur at the plate-coating stage of the assay. The inclusion of standardized controls should also provide greater consistency and data comparability among laboratories running the assay. Currently, the OEPA ELISA uses sera identified by each laboratory for positive and negative controls. Depending on which sera are available for these controls in the different locations, the definition of a positive specimen may be inconsistent. By using a defined amount of the humanized anti-IgG4 monoclonal to define the assay cutoff, results should be more comparable between laboratories and prevalence definitions should be more standardized across onchocerciasis transmission foci. Other changes included using a secondary antibody directly conjugated with HRP and the use of TMB substrate for assay development. Finally, the revised assay has an operational run time of under 90 minutes, whereas the OEPA ELISA requires almost 8 hours. Overall, these changes should result in improved ease of use, shorter assay times, and increased intralaboratory precision and interlaboratory reproducibility for onchocerciasis control program testing sites.

The onchocerciasis TPPs^24^ for MDA require a clinical diagnostic sensitivity of ≥60% for mapping and ≥89% for stopping decisions; this assay fulfills both requirements. However, both stopping and mapping use cases require specificities of ≥99.8%, which our assay’s specificity of 98.32% does not quite achieve. The OEPA ELISA has a reported specificity of 99.98% but a sensitivity of only 43%; thus failing to meet the TPP requirements as well.^13^ Using multiplex bead assays (MBA), rOv16m does achieve 99.81% specificity; however, the MBA is not readily deployable to endemic country laboratories,^21^ another requirement of the TPP. Further, reanalysis of the characteristics needed for population-based surveys suggests that no single test is likely to meet the specificity needs to verify elimination, and a combined test approach using more than one biomarker is likely necessary.^25^ In addition, efforts are underway to evaluate whether a threshold of 2% prevalence may be adequate for verifying elimination of *O. volvulus* transmission rather than the 0.1% threshold currently used by programs. If the higher threshold is adequate to interrupt transmission, this updated rOv16m ELISA would meet the test specificity needs for programmatic decision making.

As new tests are developed for various infections, our data suggest expression of antigens in mammalian cells may provide better specificity than antigens expressed in bacteria. rGST-Ov16m showed little to no cross-reactivity in the negative control or *W. bancrofti*–positive sera in immunoblots, whereas antigen expressed in *E. coli* showed faint but detectable bands (Figure 1). Curiously, we did observe potential for limited cross-reactivity with sera from some people with schistosome infections. We thought this finding may result from the GST fusion partner being an *S. japonicum* antigen. ELISAs were performed using rOv16m-GST fusion protein, rOv16m without GST, and GST alone (Table 2). Cross-reactivities observed in samples from people with schistosome infections appear to be due to anti-GST antibodies or nonspecific binding to GST rather than to rOv16m. This possible cross-reactivity is supported by their strong reactivity in the GST-only wells and absence of signal in the rOv16m-only format. By contrast, the cross-reactivity observed in one person with *A. lumbricoides* infection, in the rOv16m with and without GST assay, and negative in the GST-only format, might suggest a true antigenic cross-reaction between *A. lumbricoides* and rOv16m. This possible cross-reaction could be due to shared or homologous epitopes between rOv16m and certain *Ascaris* proteins (because rOv16m and certain *Ascaris* proteins both contain phosphatidylethanolamine-binding protein-family epitopes)^26^^,^^27^ or the polyclonal immune responses in individuals infected with *Ascaris*.^28^ Therefore, reasons for this limited cross-reactivity should be further explored.

The rOv16m-ELISA demonstrated high precision and accuracy, as well as reagent stability. Precoated plates retained activity for up to 15 months when stored at either refrigeration or room temperature. Similarly, at 37°C, the plates maintained their activity for up to 6 months. An important finding is that, even at 45°C, the plates preserved their functionality for at least 30 days. Thus, even with transport issues that may leave shipments on a hot tarmac for a few days, these data indicate that the plates will retain their reactivity.

Similarly, the humanized monoclonal antibody used for the assay standard retained activity when formulated as a contrived/spiked DBS for at least 90 days across a wide temperature range (−20°C to 37°C) and for at least 14 days at 45°C. These findings suggest that, as with plates, it is feasible to ship assay standards at ambient temperatures, in accordance with the ideal shipping criteria specified in the TPPs. Ongoing efforts have identified companies that can custom manufacture the mammalian recombinant antigen, the coated plates, and lyophilized antibody standards in a reproducible form with an associated CoA.

## CONCLUSION

We have developed an ELISA using the rOv16m antigen that demonstrates shipping stability, exhibits robust quality control, and offers rapid performance that approaches the standards set for the TPPs for onchocerciasis. Ongoing work should include additional interlaboratory reproducibility studies to ensure accuracy of the test in different endemic settings. Efforts to identify additional markers that can be used in conjunction with the rOv16m ELISA should also be pursued to achieve the very high specificity needed for programmatic use.
